# A Randomized Controlled Trial Comparing Behavioral, Educational, and Pharmacological Treatments in Youths With Chronic Tic Disorder or Tourette Syndrome

**DOI:** 10.3389/fpsyt.2018.00100

**Published:** 2018-03-27

**Authors:** Renata Rizzo, Alessandra Pellico, Paola Rosaria Silvestri, Flavia Chiarotti, Francesco Cardona

**Affiliations:** ^1^Università degli Studi di Catania, Catania, Italy; ^2^Sapienza Università di Roma, Rome, Italy; ^3^Center for Behavioral Sciences and Mental Health, Istituto Superiore di Sanità, Rome, Italy

**Keywords:** Tourette syndrome, youth, behavior therapy, pharmacological treatment, psychoeducation

## Abstract

**Context:**

The existing literature on the treatment of pediatric chronic tic disorder (CTD) and Tourette syndrome (TS) indicates that both behavioral therapy (BT) and pharmacotherapy (PT) are effective for reducing symptoms.

**Objective:**

To evaluate the efficacy of BT compared to psychoeducation (PE) or PT for reducing tics and co-occurring symptoms and for improving quality of life (QoL) in a sample of youths with CTD and TS.

**Design:**

A 10 weeks, 2 sites (Catania, Rome) randomized controlled trial. Participants were randomized to receive one of the following treatments: BT, PE, or PT.

**Participants:**

110 outpatients aged between 8 and 17 years affected by CTD or TS.

**Results:**

Patients in the BT and PT groups showed a significant reduction in the severity of tic symptoms, while the PE group did not show any improvement. PT was more effective for reducing obsessive compulsive symptoms than BT, while PE group did not show any improvement. Both BT and PT groups showed an improvement in most QoL domains, whereas no differences were found in the PE group.

**Conclusions:**

BT is as effective as pharmacological therapy in the treatment of tic disorders in children and adolescents, thus offering an alternative to medications for CTD and TS.

## Introduction

Tourette syndrome (TS) and chronic tic disorders (CTD) are neurodevelopmental disorders characterized by the presence of motor tics and/or vocal tics that occur regularly and are present for at least 12 months (1). Tic onset typically occurs at an average age of 7 years. Tics are usually preceded by premonitory urges (PU), an unpleasant sensory phenomenon that is relieved by performing tics (2). The awareness of PU increases with age (2–4) and is strongly predicted by interoceptive awareness, with higher PU known to be correlated with higher interoceptive awareness (5). TS and CTD are often comorbid with other pathologic conditions, such as obsessive-compulsive disorder (OCD), attention deficit hyperactivity disorder (ADHD), autism spectrum disorder (ASD), anxiety disorders (AD), and affective disorders (6). Several studies have shown that quality of life (QoL) is impaired in patients affected by TS and CTD, and that the presence of comorbidities is correlated with poorer perceived QoL (7, 8).

Habit reversal training (HRT) and exposure and response prevention (ERP) have demonstrated success in reducing tics (9, 10). Although there are some differences between HRT and ERP, both include the interruption of stimulus-response sequences (11). HRT is a treatment based on the detection of a competing response incompatible with tic execution, thereby physically preventing performance of the tic. This therapy is combined with other components, such as tic awareness training, relaxation training, contingency management, and generalization training (12, 13). ERP is based on the development of habituation to PU, exposing the patients to the unpleasant sensation that precedes the tics, and thus, preventing them. Both HRT and ERP [hereafter collectively referred to as behavioral therapy (BT)] offer the advantage of reducing tics without any significant adverse effects. According to the European (9) and Canadian (14) clinical guidelines and published US practice parameters (15), BT has consistently demonstrated success in reducing tic severity and is recommended as a first-line intervention for tics and TS. Whether non-pharmacological treatments should be preferred as first-line option over pharmacotherapy (PT) is a debated issue (16), and the discussion often depends not only on tic severity, but also on negative drug-induced side effects and practical aspects, including parent’s preference. However, only few studies have assessed the efficacy of BT for tic disorders and TS in childhood (see Table 1), and none of these have compared BT and other interventions to PT.

**Table 1:** Summary of studies on cognitive BT in TS patients.

Authors	Interventions	Patients (*n*°)	Mean age	Outcome measures	Results
Azrin and Peterson (27)	HR, WL	10	18.1	Tic frequency video-rating	HR > WLHR 93.5% decrease

O’Connor et al. (28)	HR/CBT > WL	47	39.1	Tic frequency video-rating	HR/CBT > WL

Wilhelm et al. (29)	HR, ST	32	36.2	Yale Global Tic Severity Scale (YGTSS)	HR > ST HR: from 30.5 to 19.8 ST: from 26.6 to 26.9

Verdellen et al. (12)	HR, ERP	43	20.6	YGTSS	HR = ERP 58% ER > 30% reduction; 28% HR > 30% reduction

Deckersbach et al. (30)	HR, ST	30	35.1	YGTSS	HR > ST HR: 39.3 to 18.3 ST: 27.7 to 26.8

Piacentini et al. (32)	HR, ST	126	11.7	YGTSS	HR > ST HR: 6 points reduction; SP: 3 points reduction

Wilhelm et al. (33)	CBT, PE, and ST	122	32	YGTSS	CBT > PE and ST

Yates et al. (31)	HRT, PE	33	10.96	YGTSS	HR: motor tic from 17.65 to 15.12; PE: motor tic 16.31 to 15.88

*HR, habit reversal; WL, waitlist; ST, supportive therapy; ERP, exposure response prevention; CBT, comprehensive behavioral therapy; PE, psychoeducation*.

This study aimed to evaluate whether BT for tics would prove superior to psychoeducation (PE) and to pharmacological treatment in reducing tics and co-occurring disorders associated with the tics in a sample of youths with TS and CTD. The study also aimed to compare the efficacy of the three treatments in improving the QoL of patients.

## Materials and Methods

### Study Design

This study was conducted across two locations, the Child and Adolescent Neuropsychiatry Units of Catania University and of Roma “La Sapienza” University. This study was performed as a controlled trial on children and adolescents with TS or CTD. Participants were randomized to receive one of three treatments: BT, PE, or PT. The study received approval from the local ethics committee of each institution (protocol numbers: Catania n. 612; Roma n. 4727). Prior to enrollment, the study personnel provided a detailed description of the study procedures and the risks and benefits to interested families, after which the parents provided written informed consent and the children and adolescents gave verbal informed assent.

### Participants

Eligible participants were patients aged 8–17 years of age with a diagnosis of TS or CTD following the DSM-5 criteria. The inclusion criteria were: tics of moderate severity as measured by the Yale Global Tic Severity Scale (YGTSS; >13 for subjects affected by TS and >9 for those affected by CTD) (17), and an intelligence quotient (IQ) >80. The exclusion criteria included epilepsy, cardiovascular disease, a family history of QT prolongation or arrhythmia, ASD, schizophrenia spectrum disorder, conduct disorder, major depression, psychosis, or addiction.

The assessment was performed by treatment-blind evaluators with extensive experience in tic disorders. Comorbid ADHD, OCD, or AD was not considered exclusion criteria unless the disorder required immediate treatment or a change in the current treatment regimen. Children who were taking anti-tic medication prior to enrollment (*n*. 6) were tapered from their treatments and underwent a 4-week washout period before the randomization and the baseline assessment. Patients randomized to BT or PE treatment were without medication during the study. Children comorbid with ADHD did not received stimulant medication during this study.

### Sample Size Determination and Children Allocation to Groups

As said above, the primary aim of the study was to analyze the effect of treatments in reducing tics and co-occurring disorders associated with the tics in a sample of youths with TS and CTD, and to assess whether BT for tics would prove superior to pharmacological treatment and PE.

Groups of 23 subjects were necessary to assess treatment effects of medium-large size (Cohen’s dz = 0.8) in reducing tics and co-occurring disorders within each treatment, using a paired Student’s *t*-test, with power = 0.80, and pairwise alpha = 0.00833 (corresponding to an experiment wise alpha = 0.05 accounting for the six comparisons: time 1 vs time 0 and time 2 vs time 0 for any of the three treatment groups).

Groups of 23 subjects were also sufficient to detect differences of large size (Cohen’s *d* = 1.1) in the change over time between treatment groups, using a Student’s *t*-test for independent groups, with power = 0.80 and pairwise alpha = 0.00833 (corresponding to an experiment wise alpha = 0.05 accounting for the six comparisons: BT vs PT, BT vs PE, and PT vs PE, either for change T1–T0 or T2–T0).

Based on the computations reported above, we decided to enroll at least 23 subjects in the BT and PE treatment groups, and at least 46 in the PT group to account for potential greater variability of the outcomes in this group due to the variety of drugs and doses administered to children. A total of 110 patients, 96 affected by TS and 14 by CTD, were randomly assigned to the groups BT (*n* = 26), PT (*n* = 57), or PE (*n* = 29), using a simple randomization plan based on a random number list and the preset unbalanced allocation ratio of1:2:1 among groups.

During the study, there was eight drop-outs: one in the group BT (low compliance), two in the group PT (low compliance), and five in the group PE (two for low compliance and three requesting to begin a drug therapy because of an increase of tic severity).

Overall, 102 patients completed the study: 25 for group BT (14 ERP, 11 HRT), 53 for PT, and 24 for PE. The average age of the participants who completed the study was 11.2 years (SD 2.43), with 79 males and 23 females.

### Interventions

#### Psychoeducation

Psychoeducation provides information about the features of the disorder and its etiology, comorbidities, and prognosis, which are explained to the children, parents, and other educators without giving advice on symptom management (9). This approach, aimed to reinforce coping strategies, reduce anxiety, and emphasize the patient’s strengths, was conducted over eight sessions. The first two sessions lasted 90 min each, then the following six sessions were 60 min each.

#### Behavioral Therapy

Behavioral therapy was conducted according to the therapist manual developed by Verdellen et al. (18). Either HRT or ERP were conducted over eight weekly sessions. Sessions were 60 min in length, although the first two sessions lasted 90 min. The primary components of HRT were tic awareness training and competing response training. In awareness training, the therapist helps the patient to detect tics, one by one, with self-monitoring. A ranking of the patient’s tics is constructed according to tic severity and level of impairment, and then the patient learns to perform a voluntary movement to physically prevent performance of the tic during the competing response training. The ERP sessions consisted of awareness training, tic detection, exposure to PU, and tic suppression. Patients were required to practice at home and parents were required to monitor tics for 15 min every day.

#### For the PT Treatment

For the PT treatment, all randomized patients were assigned to a child/adolescent psychiatrist who was responsible for their medications for the duration of the study. Drug-naïve children took risperidone at a dose ranging between 0.5 and 2 mg. Patients that had taken risperidone in the past were treated with aripiprazole, at a dose ranging between 2.5 and 10 mg. Those who had previously taken risperidone and aripiprazole were treated with pimozide, at a dose ranging between 2 and 5 mg.

### Procedures

Assessment of the patients was performed at three time points during the study, the first at baseline (T0), the second after 10 weeks (T1), and the third performed 3 months after the last session of BT or PE, or 5 months after beginning PT (T2). At each time point, patients were assessed according to YGTSS, Premonitory Urge for Tic Scale (PUTS), Diagnostic Confidence Index (DCI), Children’s Yale-Brown Obsessive-Compulsive Scale for Children (CY-BOCS), Clinical Global Impression Scale- Severity (CGI-S), and KIDSCREEN-52. At T0, the Wechsler Intelligence Scale for Children (WISC-III) was also administered. At T1 and T2, changes in tic severity were evaluated by improvement in the CGI-Improvement (CGI-I) scale.

### Measures

The Wechsler Intelligence Scale for Children (Third Edition; WISC-III) was administered to evaluate the IQ of the participants (19). The YGTSS is a clinician-rated scale, considered the gold standard in tic measurement. It consists of separate motor and vocal tic checklists scored from 0 to 5 on two subscales for motor and vocal tics. The tic dimensions assessed included the number, frequency, duration, intensity, and complexity. The subscales were combined to produce a total tic severity score (ranging from 0 to 50). Another score ranging from 0 to 50 was assigned for global impairment due to tics (17).

The TS DCI is a useful tool in clinical practice that measures the likelihood of having TS using a score from 0 to 100 (20).

The Clinical Global Impression Scale-Severity (CGI-S) and Improvement (CGI-I) are observer-rated scales that measure illness severity and global improvement or change, respectively. The severity is measured on a 7-point scale ranging from 1 to 7, in which a score of 1 represents normal and 7 are extreme. Improvement is measured on a 7-point scale that evaluates clinical changes in tics relative to the previous evaluation (T1 vs. T0 and T2 vs. T1), with a score of 1 representing very much improved, 4 representing no change and 7 indicating very much worse (21).

The Children’s Yale-Brown Obsessive-Compulsive Scale is a clinician-rated scale that assesses the type and severity of obsessive–compulsive symptoms in children. The clinician notes the presence of obsessions and compulsions, then rates the severity of these symptoms using a 0–4 score for the following categories: number, frequency, intensity, resistance, and interference (22).

The premonitory urge for Tics Scale is a self-report measure that assesses the severity of premonitory sensations (3).

KIDSCREEN-52 is a scale designed to evaluate health-related QoL of children and adolescents aged 8–18 years. KIDSCREEN contains 52 items, all scored on a 5-point scale, which measure 10 dimensions: physical well-being, psychological well-being, mood and emotions, parent relations and home life, social support and peers, school environment, social acceptance (bullying), and financial resources (23). Both a child/adolescent version and parent/proxy version were administered.

### Statistical Analysis

Statistical analyses were performed using STATA release 8.1 software. First, the distribution of quantitative variables was assessed to determine their deviation from the normal distribution within each treatment group (Shapiro–Wilk test) and the homogeneity of variance among the three treatment groups (Levene test). The baseline values of the three treatment groups for each variable were compared using the parametric analysis of variance (ANOVA) test with one grouping factor (treatment, three levels). The three treatment groups were then compared with regards to change over time for each of the variables using a mixed-model ANOVA with one between-subject factor (treatment) and one within-subject factor (time). The analysis was repeated including age at the beginning of the intervention as covariate, to account for differences in age at intervention among groups. Finally, a mixed-model ANOVA was performed on the outcome variables obtained by computing the difference between the YGTSS absolute scores at baseline (T0) and the same scores at week 10 (T1) or week 22 (T2). The analysis was repeated including age at the beginning of the intervention as covariate, to account for differences in age at intervention among groups. This analysis was also repeated including the baseline value of YGTSS or CGI-S as covariates to assess the effect of treatment on YGTSS and CGI-GI over time, taking into account the tic severity before treatment. The Tukey test was used for multiple comparisons to check whether the absolute scores differed significantly between the time points (T0 vs. T1, T0 vs. T2) for each treatment group, or if the variation in scores differed significantly between treatment groups for each time point. In addition, in order to evaluate the presence of significant changes in the CGI index as measured by CGI-I score, a one sample *t-t*est with Bonferroni correction was performed for each treatment group, and at every time point assuming a value of 4 as indicative of no change and values significantly lower or higher than 4 as indicating improvement or worsening, respectively, of the CGI over time (see Section Measures).

## Results

### Baseline

At baseline, some significant differences were found between groups; specifically, the mean scores for CGI-S and YGTSS were significantly lower in the BT group versus the PT group (*p* < 0.05 for both). Moreover, the PT group showed a lower mean score for the “mood and emotion” sub-item of KIDSCREEN-52 in comparison to the other groups (*p* < 0.05), while the PE group showed lower mean scores for the “financial resources” sub-item of the parent version of the KIDSCREEN test (*p* < 0.05; Tables 2–4).

**Table 2:** Yale Global Tic Severity Scale (YGTSS) outcome (values are expressed as absolute scores).

YGTSS	Time	BT (*n* = 25) Mean (SD)	PT (*n* = 47) Mean (SD)	PE (*n* = 24) Mean (SD)	Treatment *F* (df1, df2) *p*	Treatment × time *F* (df1, df2) *p*^#^
Motor	0 1 2	13.00 (4.03) 7.72 (3.74)* 8.52 (3.85)*	14.04 (4.07) 9.58 (3.94)* 9.09 (2.93)*	13.00 (4.46) 13.04 (4.45) 12.54 (3.90)	*F*(2.99) = 5.03 *p* = 0.0083	*F*(4,198) = 15.63 *p* < 0.0001

Phonic	0 1 2	7.08 (5.74) 3.8 (4.09)* 3.84 (4.91)*	10.28 (4.46) 6.13 (3.46)* 5.66 (3.23)*	8.92 (4.05) 8.67 (4.04) 8.54 (3.80)	*F*(2.99) = 7.23 *p* = 0.0012	*F*(4.198) = 9.60 *p* < 0.0001

Severity score	012	19.76 (8.49)11.44 (6.87)*12.36 (6.49)*	24.13 (7.50)15.70 (6.59)*14.72 (5.10)*	21.96 (7.62)21.66 (7.55)20.67 (7.38)	*F*(2.99) = 7.37 *p* = 0.0010	*F*(4.198) = 15.88 *p* < 0.0001

Global impairment	0 1 2	15.48 (11.06) 8.4 (8.43)* 7.6 (8.79)*	12.64 (11.46) 7.73 (7.76)* 7.36 (7.88)*	12.91 (10.82) 12.91 (10.83) 12.45 (10.70)	*F*(2.99) = 1.22 *p* = 0.2999	*F*(4.198) = 7.06 *p* = 0.0002

Total	0 1 2	35.4 (17.78) 19.84 (14.38)* 19.96 (13.68)*	36.38 (16.70) 23.47 (12.64)* 22.26 (11.23)*	34.25 (14.34) 35.00 (14.89) 33.12 (13.88)	*F*(2,99) = 3.22 *p* = 0.0442	*F*(4.198) = 13.51 *p* < 0.0001

*p < 0.05, time 1 or time 2 vs time 0, within each treatment group.

*^#^Greenhouse–Geisser correction for the sphericity assumption*.

**Table 3:** CGI-GI, CGI-I, Children’s Yale-Brown Obsessive-Compulsive Scale for Children (CY-BOCS), Premonitory Urge for Tic Scale (PUTS) outcome (values are expressed as absolute scores).

	Time	Behavioral therapy (BT) (*n* = 25) Mean (SD)	Pharmacotherapy (PT) (*n* = 50) Mean (SD)	Psychoeducation (PE) (*n* = 24) Mean (SD)	Treatment *F* (df1, df2) *p*	Treatment × time *F* (df1, df2)*p*^#^
CGI-GI	0 1 2	3.92 (0.99) 3.24 (0.72)* 3.20 (0.87)*	4.47 (0.77) 3.57 (0.75) * 3.51 (0.54)*	4.04 (0.86) 4.08 (0.72) 4.00 (0.66)	*F*(2.99) = 5.88 *p* = 0.0039	*F*(4.198) = 9.93 *p* < 0.0001

CGI-I^a^	0 1 2	– 2.24 (0.88)* 4.00 (1.32)	– 1.94 (0.89)* 3.74 (1.09)	– 4.00 (0.42) 3.83 (0.48)	*F*(2.99) = 30.34 *p* < 0.0001	*F*(2.99) = 15.64 *p* < 0.0001

CY-BOCS	0 1 2	8.08 (8.5) 6.60 (7.14) 5.84 (6.69)	14.36 (12.59) 10.17 (9.44)* 10.23 (9.45)*	8.29 (9.14) 8.92 (10.20) 8.38 (9.77)	*F*(2.99) = 2.49 *p* = 0.0884	*F*(4.198) = 4.53 *p* = 0.0030

PUTS	0 1 2	14.68 (7.90) 13.00 (8.46) 12.04 (8.41)*	10.89 (6.68) 9.15 (5.21)* 9.75 (6.56)	10.63 (7.57) 10.38 (6.89) 10.13 (6.90)	*F*(2.99) = 2.25 *p* = 0.1103	*F*(4.198) = 1.42 *p* = 0.2331

*p < 0.05. For all variables, but CGI-I, the comparisons are performed between time 1 or time 2 vs time 0, within each treatment group.

*^a^Asterisk refers to the significance value (with Bonferroni’s correction) of the one sample *t*-test performed within the particular treatment group and time point, assuming the value of 4 as hypothetical mean indicative of no change (see Section “Materials and Methods”). Values of CGI-I significantly lower (or higher) than 4 indicate an improvement (or worsening) of the clinical global index over time*.

*^#^Greenhouse-Geisser correction for the sphericity assumption*.

**Table 4:** KIDSCREEN-52 (patients) outcome (values are expressed as absolute scores).

KIDSCREEN52 patients sub-tests	Time	Behavioral therapy (BT) (*n* = 25) mean (SD)	Pharmacotherapy (PT) (*n* = 47) mean (SD)	Psychoeducation (PE) (*n* = 24) mean (SD)	Treatment *F* (df1, df2) *p*	Treatment × time *F* (df1, df2) *p*^#^
Physical well-being	0 1 2	19.28 (3.18) 20.04 (3.65)* 19.92 (3.59)	18.40 (5.18) 19.28 (4.58)* 20.02 (4.56)*	20.00 (4.61) 19.63 (4.78) 19.75 (4.69)	*F*(2.93) = 0.23 *p* = 0.7964	*F*(4.186) = 6.02 *p* = 0.0002

Psychological well-being	0 1 2	22.36 (3.36) 24.44 (3.58)* 23.06 (4.80)	21.34 (4.29) 21.94 (4.44) 22.72 (4.37)*	22.72 (4.37) 24.13 (3.60) 23.92 (3.57)	*F*(2.93) = 1.93 *p* = 0.1515	*F*(4,186) = 2.01 *p* = 0.1088

Moods and emotion	0 1 2	28.68 (2.87) 30.04 (2.88) 28.84 (5.44)	24.81 (5.80) 26.00 (5.93)* 27.09 (5.91)*	28.00 (6.01) 28.33 (5.12) 27.88 (5.35)	*F*(2.93) = 3.62 *p* = 0.0306	*F*(4.186) = 4.72 *p* = 0.0027

Self-perception	0 1 2	20.32 (3.70) 22.44 (2.90)* 22.36 (3.11)*	19.06 (3.90) 19.72 (3.85) 20.30 (3.72)*	20.46 (4.42) 20.96 (4.07) 21.08 (4.02)	*F*(2.93) = 2.62 *p* = 0.0780	*F*(4.186) = 4.06 *p* = 0.0108

Autonomy	0 1 2	17.72 (3.03) 19.32 (3.04)* 18.52 (4.36)	17.19 (3.99) 17.94 (3.94)* 18.15 (3.84)*	19.38 (3.88) 19.38 (3.88) 19.38 (3.88)	*F*(2.93) = 1.57 *p* = 0.2138	*F*(4.186) = 3.36 *p* = 0.0198

Social support and peers	0 1 2	23.36 (3.86) 23.56 (3.97) 22.88 (4.52)	22.32 (4.68) 22.83 (4.39) 22.94 (4.32)	22.08 (5.88) 22.29 (5.74) 21.92 (5.58)	*F*(2.93) = 0.41 *p* = 0.6651	*F*(4.186) = 1.02 *p* = 0.3959

*p < 0.05, Time 1 or Time 2 vs Time 0, within each treatment group.

*^#^Greenhouse-Geisser correction for the sphericity assumption*.

### YGTSS Outcome

**Figure 1:**
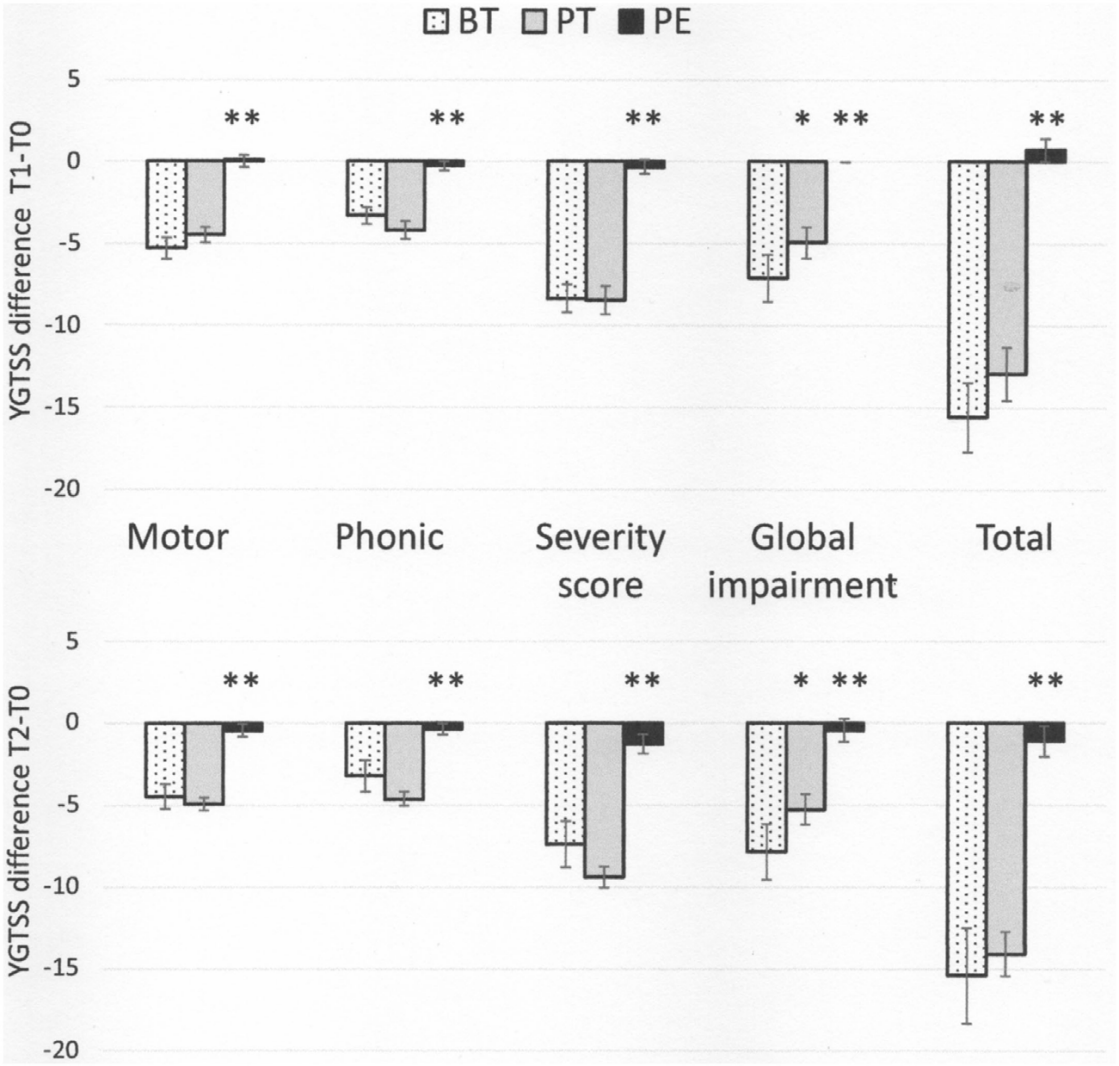
Yale Global Tic Severity Scale score variations (means and SEs) from baseline (T0) to time 1 (Tl) and 2 (T2) * *p* < 0.05, behavioral therapy (BT) vs pharmacotherapy (PT); ** *p* < 0.01, psychoeducation vs both BT and PT.

In general, patients in the BT and PT groups showed a significant reduction in the severity of tic symptoms, as assessed by YGTSS scores and sub-scores, at both T1 and T2, while the PE group did not show any improvement (Table 2). The variations in YGTSS scores are shown in Figure 1. Notably, BT and PT were equally effective in reducing YGTSS total and sub-scores, and this effect persisted over time. Both BT and PT were significantly more effective than PE in reducing YGTSS scores.

### Clinical Global Impression Scale Outcome

Patients in the BT and PT groups showed an improvement in CGI-S scores at both T1 and T2 (*p* < 0.05) and in CGI-I scores at T2 (*p* < 0.05). The PE group did not show any improvement (Table 3).

Moreover, the effect of intervention did not change when taking into account age at the beginning of intervention or tic severity at baseline expressed as YGTSS or CGI-S scores (for details, see Tables S1 and S2 in Supplementary Material).

### CY-BOCS Outcome

Pharmacotherapy significantly reduced OCD symptoms at both T1 and T2 (*p* < 0.05). The BT group reduced symptoms at both T1 and T2, but not significantly, while the PE group did not show any improvement (Table 3).

### Premonitory Urge for Tic Scale Outcome

Premonitory urges were significantly reduced by PT at T1 (*p* < 0.05) and by BT at T2 (*p* < 0.05). The PE group did not show any improvement (Table 3).

### KIDSCREEN-52 Outcome

While both BT and PT groups showed an improvement in most QoL domains, as perceived by the patients themselves, no differences in QoL were found in the PE group (Table 4). Specifically, the PT group showed an improvement in physical well-being, psychological well-being, and self-perception at T2 (*p* < 0.05), and in mood and emotions, and autonomy at both T1 and T2 (*p* < 0.05). On the other hand, BT improved physical and psychological well-being and autonomy at T1 (*p* < 0.05) and self-perception at both T1 and T2 (*p* < 0.05). Similarly, patients in the BT and PT groups showed an improvement in mean scores of QoL across many sub-scores according to feedback provided by the parents, while no differences were found in the PE group (Table 5). Parents of patients in the BT group reported improvement in their child’s QoL domains of psychological well-being, mood and emotions, self-perception and social support, and peers at both T1 and T2, and for autonomy only at T2 (*p* < 0.05 for all sub-scores). Parents of patients in the PT group reported improvement in their child’s QoL domains of psychological well-being, mood and emotions and autonomy at T2, and in physical well-being and self-perception at both T1 and T2 (*p* < 0.05 for all).

**Table 5:** KIDSCREEN-52 (parents) outcome (values are expressed as absolute scores).

KIDSCREEN-52 parents sub-tests	Time	Behavioral therapy (BT) (*n* = 25) mean (SD)	Pharmacotherapy (PT) (*n* = 47) mean (SD)	Psychoeducation (PE) (*n* = 24) mean (SD)	Treatment *F* (df1, df2) *p*	Treatment × time*F* (df1, df2) *p*^#^
Physical well-being	0 1 2	18.04 (2.88) 18.64 (3.38) 18.52 (3.60)	16.66 (4.57) 17.70 (4.43)* 19.11 (4.22)*	16.67 (4.32) 17.08 (3.94) 16.79 (4.34)	*F* (2.93) = 0.98 *p* = 0.3794	*F*(4.186) = 7.65 *p* < 0.0001

Psychological well-being	0 1 2	21.32 (3.29) 23.02 (4.36)* 23.84 (4.18)*	21.06 (5.58) 21.74 (5.47) 22.34 (5.09)*	20.54 (5.23) 20.63 (5.27) 20.25 (5.14)	*F*(2.93) = 1.39 *p* = 0. 2545	*F*(4.186) = 5.97 *p* = 0.0002

Moods and emotion	0 1 2	28.44 (3.71) 30.68 (2.67)* 30.24 (3.18)*	25.34 (9.10) 26.06 (8.99) 27.06 (8.26)*	27.83 (7.97) 28.21 (7.48) 28.33 (7.42)	*F*(2.93) = 2.11 *p* = 0.1267	*F*(4.186) = 2.90p = 0.0343

Self-perception	0 1 2	19.88 (3.28) 21.2 (3.21)* 21.44 (2.84)*	19.77 (3.74) 20.53 (3.68)* 21.21 (3.62)*	19.54 (3.97) 19.42 (4.03) 19.46 (4.00)	*F*(2.93) = 1.04 *p* = 0.3578	*F*(4.186) = 5.02 *p* = 0.0018

Autonomy	0 1 2	17.76 (3.31) 18.8 (3.11) 19.56 (3.67)*	17.89 (2.90) 18.66 (3.03) 18.74 (2.88)*	17.08 (3.75) 17.04 (3.72) 17.04 (3.72)	*F*(2.93) = 2.17 *p* = 0.1194	*F*(4.186) = 2.66 *p* = 0.0458

Social support and peers	0 1 2	19.04 (5.62) 21.88 (3.87)* 21.6 (4.88)*	18.47 (4.75) 19.26 (4.76) 19.36 (4.70)	18.08 (3.78) 18.08 (3.78) 18.00 (3.66)	*F*(2.93) = 2.81 *p* = 0.0656	*F*(4.186) = 4.60 *p* = 0.0037

*p < 0.05, time 1 or time 2 vs time 0, within each treatment group.

*^#^Greenhouse-Geisser correction for the sphericity assumption*.

## Discussion

This study investigates the efficacy of BT compared with PT and PE in reducing tics and tic-related impairment in youths with TS or CTD. The study has contributed of new findings in the research field on effects of BT for tics.

The results of this trial show that BT and PT are equally effective in reducing tic severity as measured by YGTSS total and sub-scores and by CGI-S scores. Moreover, this improvement persisted over time, at least until the end of the follow-up period (22 weeks). It is worth noting that the differences in tic severity between the three groups at baseline did not influence the efficacy of the treatments.

These results are in line with data from the literature which have highlighted the efficacy of both PT and BT in the treatment of tic disorders. Indeed, a large series of studies, including many randomized controlled trials (RCT), have previously demonstrated the efficacy of PT in the treatment of tics (24–26). BT is also a well-known treatment for patients affected by TS or tic disorders of moderate or high severity.

The first report about the efficacy of BT dates back to a paper by Azrin and Peterson (27). These authors investigated the efficacy of HRT in 10 TS patients aged 6–36 years compared with a waiting list and found that all the subjects showed substantial improvement, with a mean percent reduction in tics of 93%. Reduction occurred for both vocal and motor tics in children and adults.

O’Connor et al. (28) evaluated the efficacy of a combined treatment (awareness training, relaxation and HRT) on tic severity in 76 adult patients with CTD. The authors found a significant change in post-treatment measures, with 65% of completers reporting control over the tic ranging between 75 and 100%. There were also significant changes in measures of self-esteem, anxiety, and depression. This study compared efficacy of CBT in combination with medication and without medication.

In this study, the efficacy of BT and PT were compared with the efficacy of PE, with the first two treatments showing greater effectiveness in reducing tic severity when compared with PE.

To date, few previous studies have compared the efficacy of BT with different types of psychological treatments, and all of them found BT to be effective.

Wilhelm et al. (29) compared with a group of 32 patients with TS, the efficacy of HRT with a supportive therapy. The authors found a 10.6-point decrease in YGTSS in the group that received HRT versus no change in YGTSS in the supportive therapy group. Deckersbach et al. (30) compared the efficacy of HRT with a supportive therapy on tics. A reduction in tic severity, demonstrated by a 9.4-point decrease from the pre-treatment to mid-treatment evaluation (after eight sessions), was reported in patients who received HRT, but not in those who received supportive therapy. These results remained stable at the 6-month follow up. Both groups showed increased life satisfaction and psychosocial function.

It is worth noting that the results from our study highlighted that only BT and PT are effective treatments in improving patient QoL, whereas PE showed no effect on this dimension. This finding suggests that QoL of patients with tic disorders is a more sensitive measure to assess the patients’ global functioning than a more general index (such as life satisfaction).

To date, few studies have examined the efficacy of BT in pediatric patients. More data are available regarding the efficacy and safety in adult patients.

Yates et al. (31) performed a single blind RCT on 33 children aged 9–13 years with TS and CTD, in which they compared HRT with an educational treatment. An improvement in tic severity was found in both groups. Motor tic severity showed a greater improvement in the group that received HRT. Both groups showed a tendency toward improvement in their perceived QoL.

Piacentini et al. (32) performed a large RCT on 126 children and adolescents aged 9–17 years affected by TS or CTD, comparing BT with supportive therapy and education. The YGTSS score reduction was significantly higher in the group that received the behavioral intervention compared to the control treatment group.

Wilhelm et al. (33) compared BT for tics with PE and supportive therapy in a multisite RCT performed on 122 adults with TS (aged 16–69 years). Eight sessions of comprehensive behavioral intervention or eight sessions of supportive treatment were delivered over 10 weeks. The main outcome measures were YGTSS and CGI, which were rated by a clinician blind to treatment assignment. BT was associated with a significantly greater decrease in YGTSS (*p* < 0.001; effect size = 0.57). Also, 24 out of 63 subjects (38.1%) in the BT group were rated as having much improved or very much improved scores in the CGI-I compared to 6.8% in the control group (*p* < 0.0001). This large study on adults showed that BT is safe and effective.

McGuire et al. (34) conducted a post-treatment analysis of 248 participants enrolled in two RCT that examined the efficacy of Comprehensive Behavioral Intervention for Tics (CBIT) in reducing the severity of tic symptoms when compared to PE and supportive therapy (32, 33). The nature of bothersome tics was examined and the efficacy of CBIT for common bothersome tics was compared to PE and supportive therapy. At baseline, motor tics and tics with an urge were rated as being more bothersome than vocal tics and tics without PU. This examination suggested that CBIT outperformed supportive therapy on several tic characteristics, with the presence of a baseline urge being associated with greater tic remission for CBIT.

Only two studies have compared BT to another active behavioral intervention. Piacentini et al. (35) conducted a study on 25 children comparing HRT to awareness training with the results indicating only minimal benefit of HRT over awareness training. Verdellen et al. (12) compared HRT to ERP in a group of 43 patients affected by TS aged 7–55 years. Both treatments showed statistically significant improvement.

Woods et al. (36) conducted a *post hoc* analysis of the trial by Piacentini et al. (32) to test whether BT, in comparison to PE and supportive therapy, produced a different response on measures of other psychiatric symptoms and/or indices of psychosocial functioning. The authors found that, at 6-month post-treatment, a positive response to BT was associated with improvement in obsessive-compulsive and anxiety symptoms. In our study, BT was found to be less effective than pharmacological treatment in improving obsessive–compulsive symptoms, although this did not reach statistical significance.

To our knowledge, ours is the first study to highlight the efficacy of BT in reducing the severity of PU. PUs is unpleasant sensory phenomena that play a crucial role in triggering tics. Thus, BT may provide an additional advantage in the treatment of tic disorders by targeting both the tic symptoms themselves and the PU, and may achieve greater improvement of the patient’s global functioning.

The main limitations of this study include the short follow-up period and the small number of participants in each treatment arm. Moreover, the influence of the investigated treatments on anxiety and affective symptoms were not analyzed.

## Conclusion

This study highlights that BT is as effective as pharmacological therapy in the treatment of tic disorders in children and adolescents. Moreover, BT is effective in reducing PUs and improves the QoL of patients. When available, BT should be offered as a first-line treatment in children and adolescents with mild to moderate tic symptoms.

## Ethics Statement

This study was carried out in accordance with the recommendations of the "European clinical guidelines for Tourette Syndrome and other tic disorders. Part II: pharmacological treatment and Part III: behavioural and psychosocial interventions" with written informed consent from all subjects. All subjects gave written informed consent in accordance with the Declaration of Helsinki. The protocol was approved by the Ethic Committees of the University of Catania and Rome.

## Author Contributions

RR made a substantial contribution to the conception of the work and in writing the paper; AP, and PRS contributed to the acquisition of data; FCh contributed to statistical analysis and critically reviewed the manuscript; FCa made a substantial contribution to the conception of the work and in reviewing the paper. All the authors read and approved the final version of the manuscript.

## Conflict of Interest Statement

The authors declare that the research was conducted in the absence of any commercial or financial relationships that could be construed as a potential conflict of interest.
