# Supplementary material for: A Randomized Controlled Trial Comparing Behavioral, Educational, and Pharmacological Treatments in Youths With Chronic Tic Disorder or Tourette Syndrome
Source: Front Psychiatry. 2018 Mar 27;9:100. doi: 10.3389/fpsyt.2018.00100 (PMC5880916; doi:10.3389/fpsyt.2018.00100)
Supplement: Supplementary file 1 [file Table_1.docx]

**Tab. 1-S: YGTSS ANOVA and ANCOVA results – Three treatment groups; Three time-points**

| **YGTSS** | **ANOVA** |  |  |  | **ANCOVA** |  |
| --- | --- | --- | --- | --- | --- | --- |
|  | **Treatment**  **F (df1, df2)**  **p** | **Treatment x Time**  **F (df1, df2)**  **p**^#^ |  | **Treatment**  **F (df1, df2)**  **p** | **Treatment x Time**  **F (df1, df2)**  **p**^#^ | **Age**  **F (df1, df2)**  **p** |
| **Motor** | F(2,99) = 5.03  p = 0.0083 | F(4,198) = 15.63  p < 0.0001 |  | F(2,98) = 4.87  p = 0.0097 | F(4,198) = 15.63  p < 0.0001 | F(1,98) = 0.65  p = 0.4214 |
| **Phonic** | F(2,99) = 7.23  p = 0.0012 | F(4,198) = 9.60  p < 0.0001 |  | F(2,98) = 8.02  p = 0.0006 | F(4,198) = 9.60  p < 0.0001 | F(1,98) = 0.04  p = 0.8434 |
| **Severity Score** | F(2,99) = 7.37  p = 0.0010 | F(4,198) = 15.88  p < 0.0001 |  | F(2,98) = 7.62  p = 0.0008 | F(4,198) = 15.88  p < 0.0001 | F(1,98) = 0.11  p = 0.7380 |
| **Global Impairment** | F(2,99) = 1.22  p = 0.2999 | F(4,198) = 7.06  p = 0.0002 |  | F(2,98) = 1.22  p = 0.2985 | F(4,198) = 7.06  p = 0.0002 | F(1,98) = 0.00  p = 1.0000 |
| **Total** | F(2,99) = 3.22  p = 0.0442 | F(4,198) = 13.51  p < 0.0001 |  | F(2,98) = 3.31  p = 0.0407 | F(4,198) = 13.51  p < 0.0001 | F(1,98) = 0.48  p = 0.4893 |

ANOVA model: Between-subject factor = Treatment (BT vs PT vs PE), Repeated measures factor = Time (0 vs 1 vs 2)

ANCOVA model: Between-subject factor = Treatment (BT vs PT vs PE), Repeated measures factor = Time (0 vs 1 vs 2), Covariate = Age at the beginning of treatment

^#^ Greenhouse-Geisser correction for the sphericity assumption
