# Supplementary material for: A Randomized Controlled Trial Comparing Behavioral, Educational, and Pharmacological Treatments in Youths With Chronic Tic Disorder or Tourette Syndrome
Source: Front Psychiatry. 2018 Mar 27;9:100. doi: 10.3389/fpsyt.2018.00100 (PMC5880916; doi:10.3389/fpsyt.2018.00100)
Supplement: Supplementary file 2 [file Table_2.docx]

**Tab. 2-S: YGTSS ANOVA and ANCOVA results – Three treatment groups; Two variations, from baseline to Time 1 or Time 2**

| **YGTSS** | **ANOVA** |  |  |  | **ANCOVA** |  |
| --- | --- | --- | --- | --- | --- | --- |
|  | **Treatment**  **F (df1, df2)**  **p** | **Treatment x Time**  **F (df1, df2)**  **p** |  | **Treatment**  **F (df1, df2)**  **p** | **Treatment x Time**  **F (df1, df2)**  **p** | **YGTSS score**  **F (df1, df2)**  **p** |
| **Motor** | F(2,99) = 26.76  p < 0.0001 | F(2,99) = 2.04  p = 0.1358 |  | F(2,98) = 34.79  p < 0.0001 | F(2,99) = 2.04  p = 0.1358 | F(1,98) = 0.00  p = 1.0000 |
| **Phonic** | F(2,99) = 15.75  p < 0.0001 | F(2,99) = 0.33  p = 0.7209 |  | F(2,98) = 27.18  p < 0.0001 | F(2,99) = 0.33  p = 0.7209 | F(1,98) = 0.26  p = 0.6106 |
| **Severity Score** | F(2,99) = 27.51  p < 0.0001 | F(2,99) = 1.54  p = 0.2193 |  | F(2,98) = 41.67  p < 0.0001 | F(2,99) = 1.54  p = 0.2193 | F(1,98) = 0.09  p = 0.7645 |
| **Global Impairment** | F(2,99) = 9.12  p = 0.0002 | F(2,99) = 0.11  p = 0.9002 |  | F(2,98) = 12.81  p < 0.0001 | F(2,99) = 0.11  p = 0.9002 | F(1,98) = 0.56  p = 0.4549 |
| **Total** | F(2,99) = 20.36  p < 0.0001 | F(2,99) = 0.41  p = 0.6615 |  | F(2,98) = 32.45  p < 0.0001 | F(2,99) = 0.41  p = 0.6615 | F(1,98) = 0.02  p = 0.8825 |

ANOVA model: Between-subject factor = Treatment (BT vs PT vs PE), Repeated measures factor = Variation (1-0 vs 2-0)

ANCOVA model: Between-subject factor = Treatment (BT vs PT vs PE), Repeated measures factor = Variation (1-0 vs 2-0), Covariate = YGTSS score (the same as the outcome) at the beginning of treatment
